# Anticancer sensitivities and biological characteristics of HCT116 cells resistant to the selective poly(ADP‐ribose) glycohydrolase inhibitor

**DOI:** 10.1002/2211-5463.70178

**Published:** 2025-12-05

**Authors:** Kaede Tsuda, Yoko Ogino, Akira Sato

**Affiliations:** ^1^ Department of Biochemistry and Molecular Biology, Faculty of Pharmaceutical Sciences Tokyo University of Science Tokyo Japan

**Keywords:** ARH3, NAMPT, PAR, PARG, PARP, PDD00017273

## Abstract

Poly(ADP‐ribose) glycohydrolase (PARG) is a key enzyme involved in poly(ADP‐ribose) (PAR) degradation and is considered a potential anticancer target. We previously investigated resistance mechanisms to the PARG inhibitor PDD00017273 in human colorectal cancer HCT116 cells and established an acquired PDD00017273‐resistant HCT116R^PDD^ cell line. In this study, we analyzed the protein levels of enzymes associated with PAR metabolism in both parental HCT116 cells and resistant HCT116R^PDD^ cells using western blotting. PARG expression levels were similar between HCT116R^PDD^ and HCT116 cells. However, the levels of PARP1 and ARH3 were reduced in HCT116R^PDD^ cells compared to HCT116 cells. Nevertheless, intracellular PAR levels were elevated in HCT116R^PDD^ cells. Interestingly, HCT116R^PDD^ cells exhibited greater sensitivity to γ‐ray irradiation and the nicotinamide phosphoribosyltransferase (NAMPT) inhibitor FK866 than the parental HCT116 cells, yet showed comparable sensitivity to 5‐FU, cisplatin, and PARP inhibitors olaparib, talazoparib, and veliparib. Furthermore, we observed that HCT116R^PDD^ cells tended to maintain slightly higher levels of intracellular NAD^+^/NADH and ATP compared to parental HCT116 cells. These findings suggest that cancer cells employ a mechanism to regulate NAD^+^ and ATP levels, thereby avoiding cell death from intracellular PAR accumulation through coordinated PARP–PARG regulation.

## Graphical abstract

We analyzed alterations of PAR metabolism‐related proteins in PARG inhibitor‐resistant HCT116R^PDD^ cells. Although PARG levels remained unchanged, HCT116R^PDD^ cells exhibited reduced PARP1 and ARH3 expression and elevated PAR levels. Interestingly, HCT116R^PDD^ cells exhibited slightly elevated intracellular NAD^+^/NADH and ATP levels. Our findings suggest a novel resistance mechanism involving coordinated PARP–PARG regulation and metabolic adaptation.

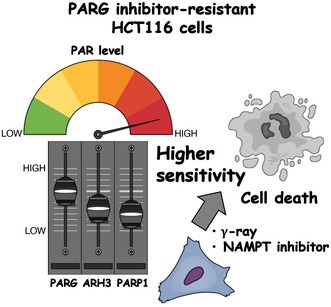

**Abbreviations**
ARH3: ADP‐ribosyl hydrolase 3
NAMPT: nicotinamide phosphoribosyltransferase
PAR: poly(ADP‐ribose)
PARG: poly(ADP‐ribose) glycohydrolase
PARP: poly(ADP‐ribose) polymerase

Poly(ADP‐ribosyl)ation (PARylation) is a reversible posttranslational modification mediated by poly(ADP‐ribose) polymerases (PARPs), primarily PARP1 and PARP2, and subsequently resolved by poly(ADP‐ribose) glycohydrolase (PARG) [1, 2, 3, 4, 5]. Poly(ADP‐ribose) (PAR), added to target proteins, mainly PARPs themselves and DNA repair proteins by PARPs using nicotinamide adenine dinucleotide (NAD^+^) as a substrate, is eventually degraded into ADP‐ribose by PARG and ADP‐ribosyl hydrolase 3 (ARH3) [1, 2, 3, 4]. Among these, PARP and PARG—key regulators of PARylation—have attracted significant attention as targets for anticancer drug development [4, 5]. Indeed, selective PARP inhibitors such as olaparib, talazoparib, and veliparib have been successfully developed [5, 6]. In contrast, despite the identification of numerous natural and synthetic compounds as potential PARG inhibitors, issues related to their selectivity, potency, and cell permeability remain significant challenges [4, 6]. The PARG inhibitor PDD00017273 selectively and effectively inhibits PARG without affecting PARP1 or ARH3 activity [5, 6, 7]. Moreover, PDD00017273 is the first reported inhibitor to demonstrate effective cell permeability and activity at the cellular level [5, 7]. Numerous studies utilizing PDD00017273 have elucidated the molecular functions of PARG in DNA repair, cell differentiation, cell death, and tumor biology [8, 9, 10, 11].

NAD^+^ is an essential redox cofactor critical to energy metabolism, especially in glycolysis, the TCA cycle, and oxidative phosphorylation [4, 12]. Additionally, it functions as a substrate for various enzymes, including sirtuins and PARPs, which are involved in cellular signaling, calcium homeostasis, gene expression, genome stability, and decisions governing cell survival or death [4, 13, 14, 15, 16]. NAD^+^ is synthesized via the *de novo* pathway from tryptophan and two salvage pathways from nicotinamide (NAM) and nicotinic acid (NA) [4, 17]. Many cancer cells exhibit elevated NAD^+^turnover and predominantly rely on the NAM salvage pathway. Nicotinamide phosphoribosyltransferase (NAMPT), the rate‐limiting enzyme of this pathway, has emerged as a promising target for anticancer therapy [12, 18, 19]. FK866 (also known as APO866 or WK175), a potent and specific NAMPT inhibitor, has been evaluated in clinical trials as a potential chemotherapeutic agent [20, 21].

Previously, we established the PDD00017273‐resistant HCT116R^PDD^ cell line from the parental human colorectal cancer HCT116 cells using the selective PARG inhibitor PDD00017273 [22]. We also reported that HCT116R^PDD^ cells carry specific mutations in PARG (Glu352Gln) and PARP1 (Lys134Asn) [22]. Furthermore, while PARG protein levels remained comparable between HCT116R^PDD^ and HCT116 cells, PARP1 protein levels were significantly reduced in HCT116R^PDD^ cells relative to the parental HCT116 line [22]. We hypothesize that qualitative changes in PARG, along with both qualitative and quantitative changes in PARP, contribute to resistance against the selective PARG inhibitor PDD00017273.

In this study, we investigated PARylation status and the expression of PAR metabolism‐related proteins in both parental HCT116 cells and the PDD00017273‐resistant HCT116R^PDD^ cells. Additionally, we evaluated the sensitivity of both cell lines to the anticancer agents 5‐fluorouracil (5‐FU) and cisplatin, γ‐ray irradiation, PARP inhibitors—olaparib, talazoparib, and veliparib—as well as the NAMPT inhibitor FK866. We further examined NAMPT activity and intracellular levels of NAD^+^/NADH and ATP in HCT116R^PDD^ and parental HCT116 cells. Finally, we discuss potential mechanisms driving resistance to PDD00017273 and enhanced sensitivity to γ‐ray irradiation and FK866 in the context of altered expression of PARylation and PAR metabolism‐related proteins.

## Methods

### Reagents

The selective PARG inhibitor PDD00017273 and the specific PARP inhibitors talazoparib and veliparib were purchased from MedChemExpress (Monmouth Junction, NJ, USA). The PARP inhibitor olaparib was obtained from Cayman Chemical (Ann Arbor, MI, USA), and the NAMPT inhibitor FK866 was sourced from Focus Biomolecules. The anticancer drugs 5‐FU and CDDP were purchased from FUJIFILM Wako Pure Chemical Corporation (Osaka, Japan). PDD00017273, talazoparib, veliparib, olaparib, and 5‐FU were prepared as 100 mm stock solutions in dimethyl sulfoxide (DMSO; Sigma‐Aldrich, Merck KGaA, Darmstadt, Germany) and stored at −20 °C. CDDP was freshly dissolved in DMSO immediately before use. FK866 hydrochloride was prepared as a 10 mm stock solution in ultrapure water and stored at −20 °C.

### Cell culture

The human colon cancer cell line HCT116 (RRID: CVCL_0291) was obtained from the American Type Culture Collection (Manassas, VA, USA). The PARG inhibitor‐resistant cell line HCT116R^PDD30^ was generated following a previously established protocol [22]. Both parental and PDD00017273‐resistant HCT116 cells were cultured in DMEM medium (FUJIFILM Wako Pure Chemical Corporation) supplemented with 10% heat‐inactivated fetal bovine serum (Capricorn Scientific, Ebsdorfergrund, Germany), 100 units·mL^−1^ penicillin, and 100 μg·mL^−1^ streptomycin (FUJIFILM Wako Pure Chemical Corporation) in a 37 °C incubator maintained at 5% CO_2_ and 100% relative humidity. Cell viability was assessed using a hemocytometer and trypan blue dye exclusion. All experiments were performed with mycoplasma‐free cells.

### Colony formation assay

Colony formation assays were conducted as described previously [22, 23, 24]. HCT116 and HCT116R^PDD^ cells were dissociated using Accutase, resuspended in medium, seeded into 6‐well plates (200 cells per well) in duplicate, and incubated overnight. Cells were then treated with various drug concentrations or solvent controls (DMSO or water). After ten days of incubation, colonies were fixed in 4% formaldehyde, stained with 0.1% (w/v) crystal violet, and counted. The colony formation rate at each concentration of each drug was expressed as the relative value of the number of colonies formed by drug‐treated cells compared to the number of colonies in the control group, which was set at 100%. The 50% effective concentration (EC_50_) was determined from the colony formation curve.

### Gamma‐ray irradiation

Gamma‐ray irradiation was conducted as previously described [23]. HCT116 and HCT116R^PDD^ cells were seeded into 6‐well plates (200 cells per well) in duplicate and incubated overnight. The cells were then exposed to gamma‐ray irradiation at 2, 4, 6, or 8 Gy using a Cs‐137 Gammacells 40 Exactor (Best Theratronics, Ltd., Kanata, ON, Canada) with a dose rate of 0.687 Gy·min^−1^ and subsequently subjected to colony formation assays.

### Western blot analysis

Western blot analysis was performed as described previously [22, 24] using the following antibodies: mouse anti‐PARG (D8B10) monoclonal antibody (1:500; MABS61, Sigma‐Aldrich), rabbit anti‐PARP polyclonal antibody (1:1000; 9542, Cell Signaling Technologies, Danvers, MA, USA), mouse anti‐poly(ADP‐ribose) monoclonal antibody 10H (1:500; Cat#ALX‐804‐220, Enzo Life Sciences), mouse anti‐ARH3 (A‐7) antibody (1:100; sc‐374 162, Santa Cruz Biotechnologies), rat anti‐NAMPT clone 14A.5 (1:1000; MABS465, Merck), mouse anti‐H2AX monoclonal antibody (1:1000; 05–636, Merck), rabbit anti‐H2AX polyclonal antibody (1:5000; A300‐082‐A, Bethyl Technologies), mouse monoclonal anti‐beta actin clone AC‐15 (1:20000; A1978, Sigma‐Aldrich), horseradish peroxidase (HRP)‐linked anti‐rat IgG (1:20000; NA935, GE Healthcare, Marlborough, MA, USA), HRP‐linked anti‐rabbit IgG (1:20000; NA934V, GE Healthcare), and HRP‐linked whole antibody anti‐mouse IgG (1:20000; NA931V, GE Healthcare).

### Analysis of NAMPT activity

The NAMPT activity assay was performed as described previously [24]. Whole‐cell lysates were prepared by incubating cells in lysis buffer (2% CHAPS in PBS with a protease inhibitor cocktail [Roche]) on ice for 30 min. Lysates were then sonicated using a Branson Sonifier‐250 and centrifuged at 15 000 ***g*** for 15 min at 4 °C. The resulting supernatants, containing the solubilized protein fractions, were used for enzymatic assays. NAMPT activity was measured using a coupled‐enzyme reaction system (CycLex NAMPT colorimetric assay kit) in a 96‐well plate format, following the one‐step method.

### Measurement of intracellular NAD ^+^/NADH levels

Intracellular NAD^+^/NADH levels were measured as previously described [24]. Parental HCT116 and HCT116R^PDD^ cells were dissociated using Accutase, suspended in PBS, and cell suspensions (7500 cells·tube^−1^) were subjected to NAD^+^/NADH assays using the NAD/NADH‐Glo Assay kit (Promega). Luminescent signals were then measured using a Tecan microplate reader.

### Measurement of intracellular ATP levels

For intracellular ATP level measurement, parental HCT116 and HCT116R^PDD^ cells were dissociated with Accutase and suspended in PBS. Cell suspensions (5000 cells·well^−1^) were used in the CellTiter‐Glo® 2.0 Cell Viability Assay (Promega, WI, USA). Luminescence was recorded using a Tecan microplate luminometer (Mannedorf, Switzerland).

### Statistical analysis

Statistical analyses were performed using GraphPad Prism 9 software. Data are presented as mean ± standard error. Significant differences among groups were determined using Student's *t*‐test. Values of *P < 0.05, **P < 0.01 and ***P < 0.001 were considered statistically significant.

## Results

### Status of PARylation and PAR metabolism‐related enzymes PARG, PARP, and ARH3 in parental HCT116 cells and PDD00017273‐resistant HCT116R^PDD^ cells

**Fig. 1:**
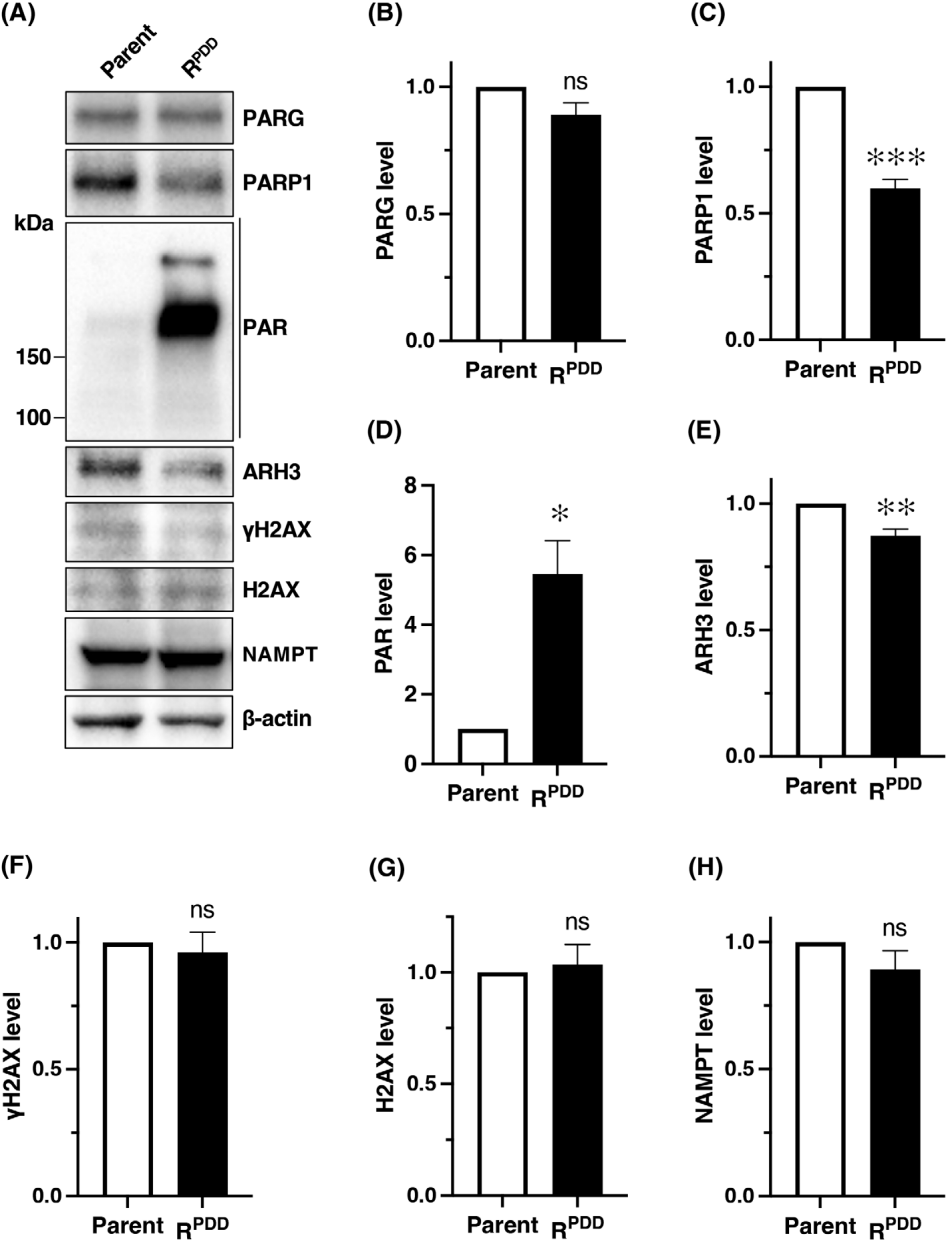
The levels of PARylation and poly(ADP‐ribose) metabolism‐related enzymes in parental HCT116 cells and PDD00017277‐resistant HCT116R^PDD^ cells. Under untreated conditions, whole‐cell lysates were prepared from HCT116 and HCT116R^PDD^ cells (without PDD00017273 treatment). Protein levels of PARG, PARP1, PAR, ARH3, γH2AX, H2AX, NAMPT, and β‐actin were assessed via western blot analysis. β‐actin was used as an internal loading control. The data represent three independent experiments. The levels of (B) PARG, (C) PARP1, (D) PAR, (E) ARH3, (F) γH2AX, (G) H2AX, and (H) NAMPT in parental HCT116 and HCT116R^PDD^ cells are shown. Protein expression in HCT116R^PDD^ cells is presented as the ratio of each protein band's density relative to that in parental HCT116 cells. Values represent the mean of three independent experiments, with error bars indicating ± SE from triplicate (*n* = 3). Student's *t*‐test, ns, no significant difference. *P < 0.05, **P < 0.01, ***P < 0.001.

We developed a PDD00017273‐resistant human colorectal cancer cell line, HCT116R^PDD^, from parental HCT116 cells to investigate mechanisms underlying resistance to the selective PARG inhibitor and to identify vulnerabilities in the resistant cells (Table 1) [22]. This study examined the status of proteins involved in PARylation in HCT116R^PDD^ cells and their sensitivity to anticancer drugs, radiation, and inhibitors of PARP and NAMPT. First, we analyzed intracellular PARylation levels and the expression of PAR metabolism‐associated proteins in resistant HCT116R^PDD^ and parental HCT116 cells (Fig. 1), using β‐actin as an internal control. As reported previously [22], PARG protein levels were comparable between HCT116R^PDD^ and parental HCT116 cells (Fig. 1A,B), while PARP levels were reduced in HCT116R^PDD^ cells (Fig. 1A,C). Conversely, PARylation levels were elevated in the resistant HCT116R^PDD^ cells (Fig. 1A,D). Notably, ARH3 expression was decreased in HCT116R^PDD^ cells compared to the parental line (Fig. 1A,E). In contrast, the protein levels of γH2AX, total H2AX, and NAMPT were similar between the resistant and parental HCT116 cells (Fig. 1A,F–H).

**Table 1:** The sensitivities of specific poly(ADP‐ribose) glycohydrolase (PARG) inhibitor PDD00017273 in parental HCT116 cells and the resistant HCT116R^PDD^ cells. N.D., not detected.

	EC_50_ (μm)	
	HCT116	HCT116R
PDD00017273	60.0 ± 17.6	N.D. (>100.0)

### Anticancer effects of the anticancer medicines 5‐FU, CDDP, and radiation in parental HCT116 cells and PDD00017273‐resistant HCT116R^PDD^ cells

**Fig. 2:**
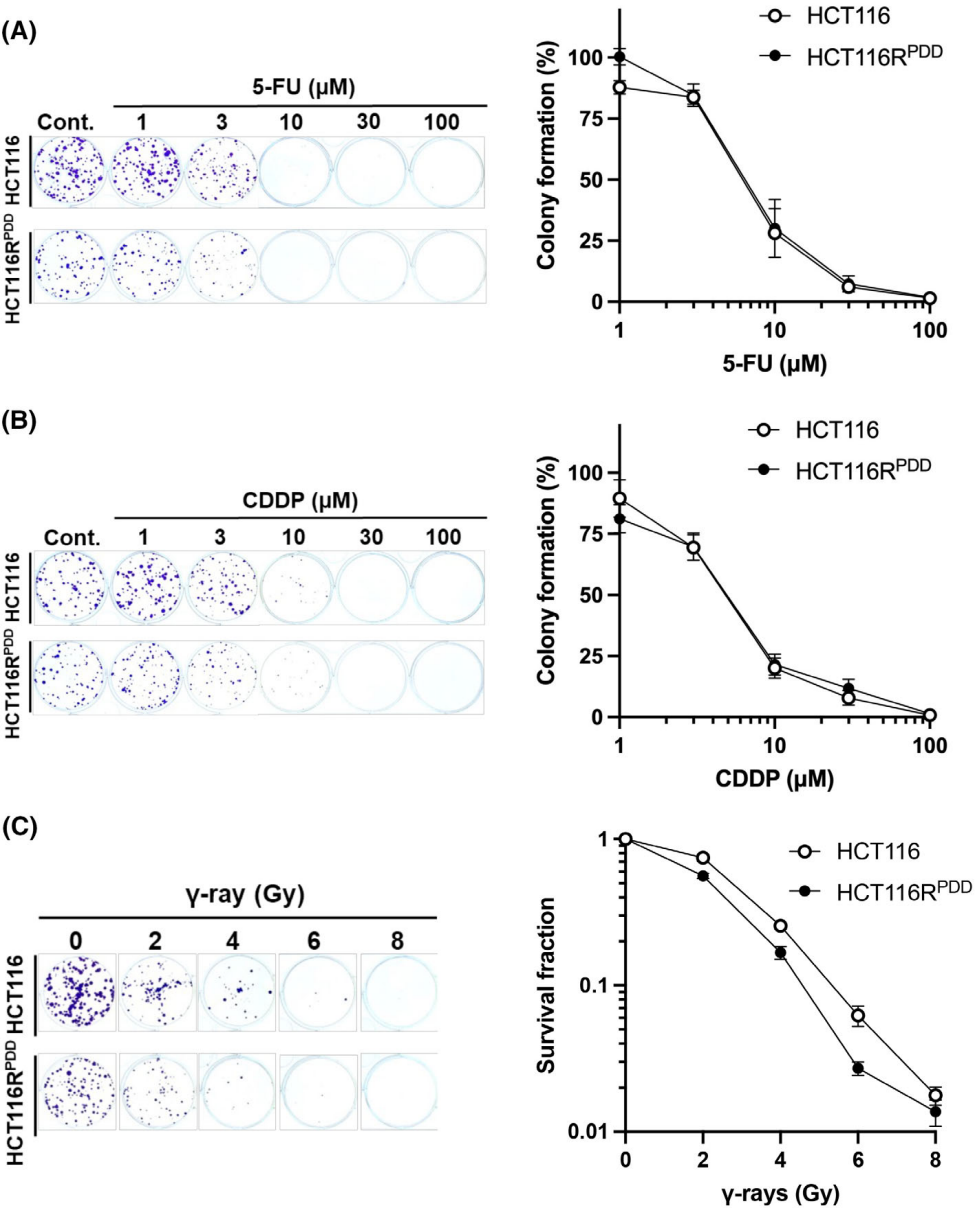
The anticancer effect of the HCT116 and HCT116R^PDD^ cells on the anticancer drugs, 5‐FU and CDDP, and γ‐ray irradiation. HCT116 and HCT116R^PDD^ cells were treated with the indicated concentrations of (A) 5‐FU, (B) CDDP, or with the indicated dose of (C) γ‐ray, followed by incubation for 10 days. Colony formation and survival fraction represent the mean of two independent experiments, each performed in duplicate, with error bars indicating ±SE from four measurements (*n* = 4). White circles represent HCT116 cells, and black circles represent HCT116R^PDD^ cells.

We investigated the effects of various anticancer agents, including 5‐fluorouracil (5‐FU). CDDP and γ‐ray irradiation on the proliferation of both parental HCT116 cells and PDD00017273‐resistant HCT116R^PDD^ cells using a colony formation assay (Fig. 2). As shown in Fig. 2A,B, and Table 2, the resistance indices for 5‐FU and CDDP were 1.1‐fold (EC_50_ = 7.4 μm in HCT116R^PDD^ cells; EC_50_ = 6.9 μm in HCT116 cells) and 1.0‐fold (EC_50_ = 5.0 μm in HCT116R^PDD^ cells; EC_50_ = 4.9 μm in HCT116 cells), respectively. In contrast, the 37% sensitizer enhancement ratio (SER_37_) for γ‐ray irradiation was reduced by 0.8‐fold in HCT116R^PDD^ cells (SER_37_ = 2.7) compared with the parental HCT116 cells (SER_37_ = 3.3) (Fig. 2C and Table 3). These findings suggest that HCT116R^PDD^ cells, though resistant to PDD00017273, are more sensitive to γ‐ray irradiation but exhibit comparable sensitivity to 5‐FU and CDDP relative to parental HCT116 cells.

**Table 2:** Effects of 5‐FU and CDDP in parental HCT116 cells and PDD00017273‐resistant HCT116R^PDD^ cells.

	EC_50_ (μm)	
	HCT116	HCT116R
5‐FU	6.9 ± 1.3	7.4 ± 1.8
CDDP	4.9 ± 0.6	5.0 ± 0.7

**Table 3:** Sensitivity to γ‐ray irradiation in parental HCT116 cells and PDD00017273‐resistant HCT116R^PDD^ cells.

	SER_37_	
	HCT116	HCT116R
γ‐ray irradiation	3.3	2.7

### Anticancer sensitivities of selective PARP inhibitors in parental HCT116 cells and PDD00017273‐resistant HCT116R^PDD^ cells

**Fig. 3:**
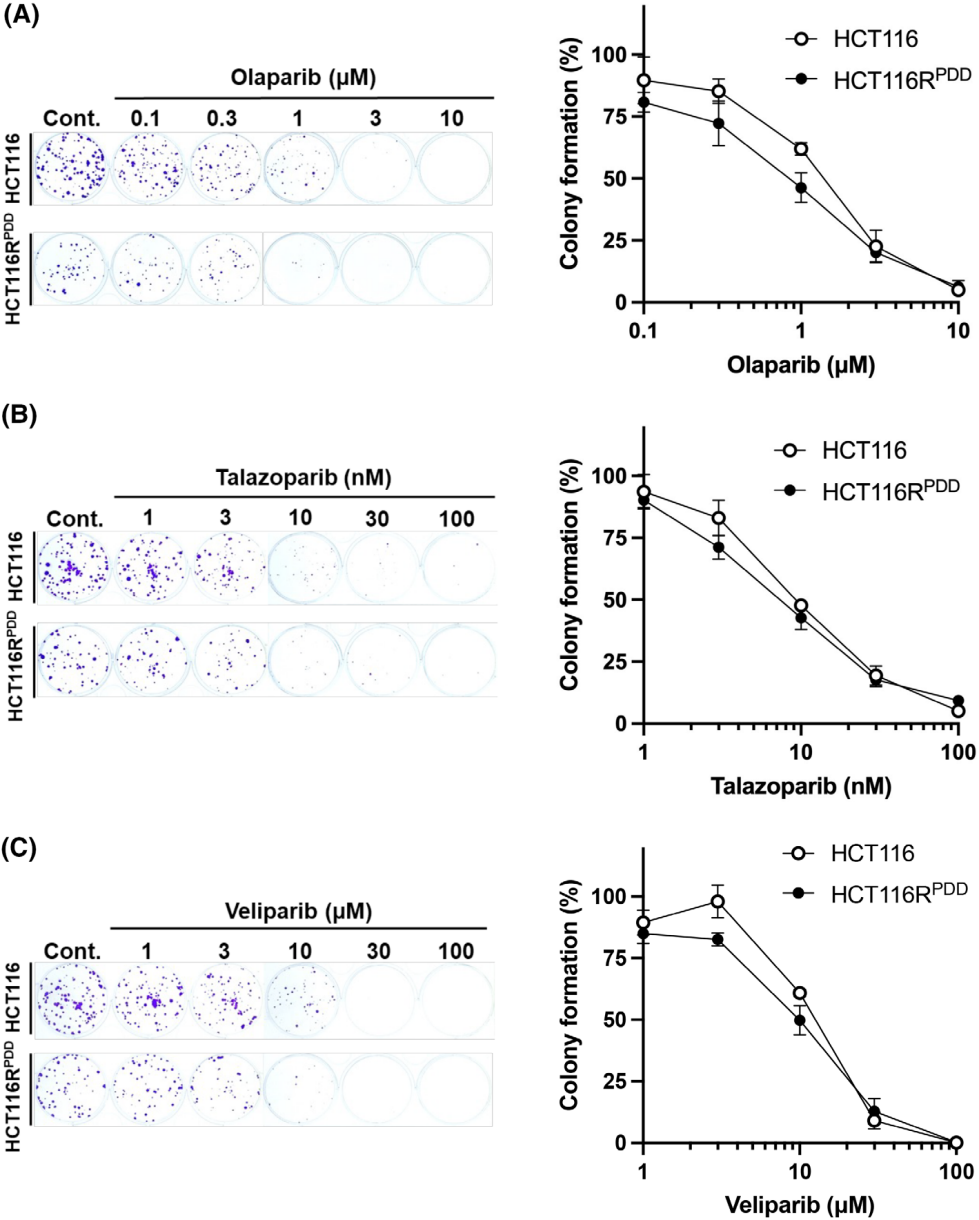
The anticancer sensitivity of the HCT116 and HCT116R^PDD^ cells to several specific PARP inhibitors, Olaparib, talazoparib, and veliparib. Drug sensitivity of HCT116 and HCT116R^PDD^ cells was assessed using the colony formation assay. Cells were treated with the indicated concentrations of (A) Olaparib, (B) Talazoparib, and (C) Veliparib and incubated for 10 days. Colony formation represents the average of two independent experiments, each conducted in duplicate, with error bars indicating ±SE from four measurements (*n* = 4). White circles indicate HCT116 cells; black circles indicate HCT116R^PDD^ cells.

We then assessed the sensitivity of selective PARP inhibitors, including olaparib, talazoparib, and veliparib, in both parental HCT116 and HCT116R^PDD^ cells (Fig. 3). The resistant HCT116R^PDD^ cells displayed similar sensitivity to PARP inhibitors as the parental HCT116 cells (Fig. 3A–C and Table 4). A closer examination revealed that HCT116R^PDD^ cells tended to be more responsive at lower concentrations of each PARP inhibitor compared to the parental cells (Fig. 3A–C). These findings indicate that HCT116R^PDD^ cells show similar responses to PARP inhibitors olaparib, talazoparib, and veliparib.

**Table 4:** Sensitivities to the selective poly(ADP‐ribose) polymerase (PARP) inhibitors olaparib, talazoparib, and veliparib and the specific nicotinamide phosphoribosyltransferase (NAMPT) inhibitor FK866 in parental HCT116 cells and PDD00017273‐resistant HCT116R^PDD^ cells.

	EC_50_	
	HCT116	HCT116R
Olaparib (μm)	1.5 ± 0.2	1.0 ± 0.1
Talazoparib (nm)	8.8 ± 0.6	7.5 ± 1.3
Veliparib (μm)	12.3 ± 0.3	9.8 ± 1.2
FK866 (nm)	13.5 ± 1.5	5.6 ± 0.4

### Status of NAMPT activity, intracellular NAD ^+^/NADH, and ATP levels in parental HCT116 cells and PDD00017273‐resistant HCT116R^PDD^ cells

**Fig. 4:**
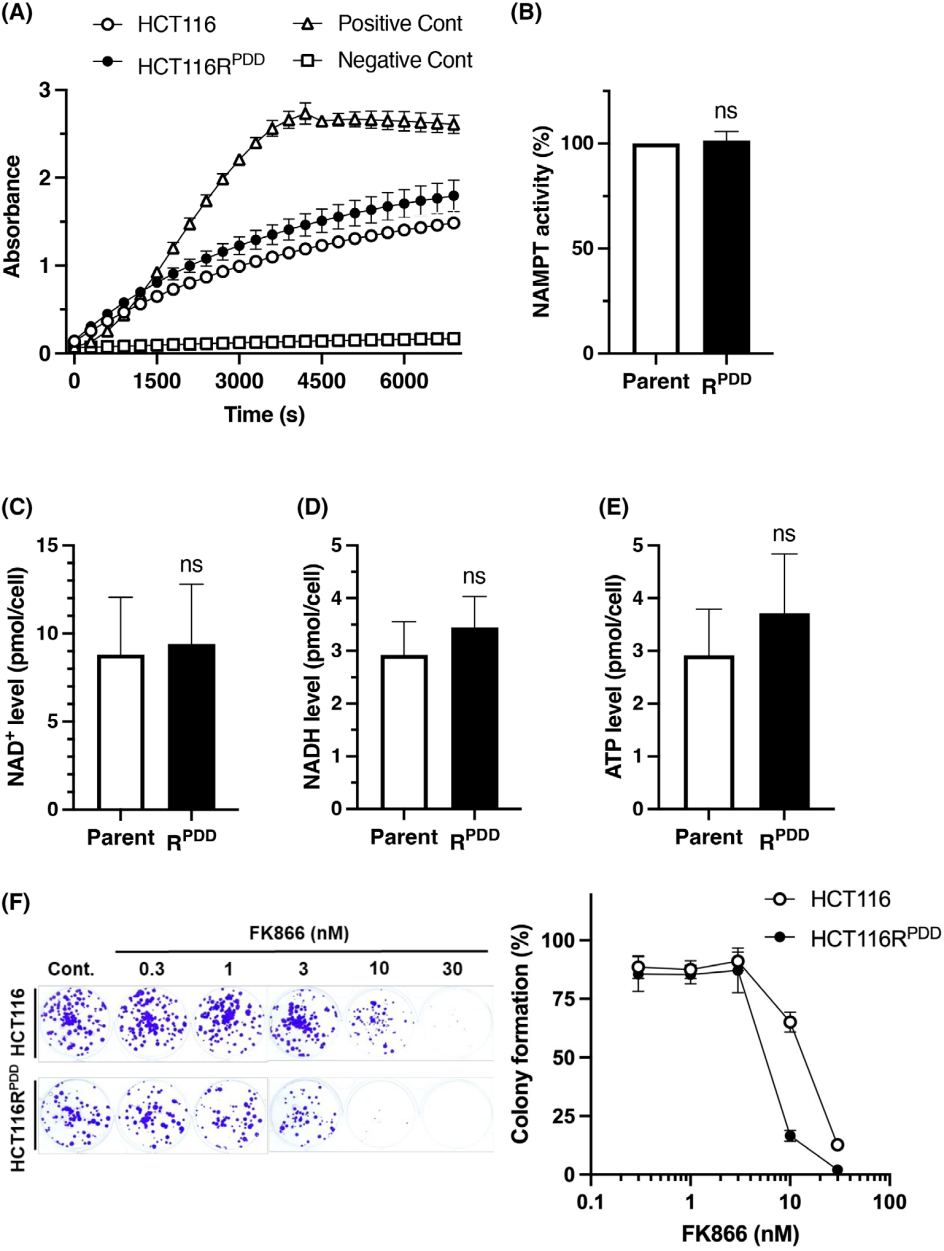
The NAMPT enzyme activity and the levels of intracellular NAD^+^/NADH and ATP in HCT116 and HCT116R^PDD^ cells. (A) NAMPT activity in HCT116R^PDD^ and HCT116 cells was assessed using the Cyclex NAMPT colorimetric assay kit. Absorbance at 450 nm was recorded every 5 min for 2 h. The r‐values represent the average of three independent experiments, each conducted in triplicate, with error bars showing ±SE from three measurements (*n* = 3). The positive control refers to the recombinant NAMPT group, while the negative control refers to the lysis buffer‐only group. (B) NAMPT activity was calculated by dividing the change in absorbance within the linear range by the corresponding reaction time. The NAMPT activity of HCT116R^PDD^ cells is shown as a ratio relative to that of HCT116 cells. (C) NAD^+^, (D) NADH, and (E) ATP levels in HCT116 and HCT116R^PDD^ cells. NAD^+^ (pmol cell^−1^) and NADH (pmol cell^−1^) levels represent the mean of three independent experiments, each performed in triplicate, with error bars showing ±SE from nine values (*n* = 9). ATP level (pmol cell^−1^) also represents the mean of three independent experiments, each conducted in triplicate, with error bars showing ±SE from nine values (*n* = 9). Student's *t*‐test, no significant difference. (F) Colony formation by HCT116R^PDD^ cells and parental HCT116 cells after 10 days of treatment with FK866. Colony formation represents the mean of two independent experiments, each performed in duplicate, with error bars indicating ±SE from four measurement values (*n* = 4).

To investigate the role of NAMPT in HCT116R^PDD^ and HCT116 cells, we assessed NAMPT enzymatic activity in whole‐cell lysates from both cell lines. As shown in Fig. 4B,C, NAMPT activity levels in HCT116R^PDD^ and HCT116 cells were nearly identical. It is widely recognized that the regulation of intracellular PAR levels is vital for cellular viability, as an excessive accumulation of PAR can lead to the depletion of NAD^+^ and interfere with ATP production [25, 26, 27]. Therefore, we subsequently measured intracellular NAD^+^/NADH and ATP levels in both HCT116R^PDD^ and HCT116 cells. The intracellular NAD^+^/NADH and ATP levels were slightly elevated in HCT116R^PDD^ cells compared to the parental HCT116 cells (Fig. 4C–E). These findings suggest that although NAMPT activity is comparable, the intracellular NAD^+^/NADH and ATP levels tend to be slightly higher in the HCT116R^PDD^ cells. Additionally, we examined the sensitivity to the NAMPT inhibitor FK866 in both cell types. Interestingly, the HCT116R^PDD^ cells were more sensitive to FK866 than the parental HCT116 cells (Fig. 4F and Table 4). These results indicate that, despite similar protein levels and enzyme activity of NAMPT in both cell types, HCT116R^PDD^ cells are more sensitive to the NAMPT inhibitor FK866 compared to the parental HCT116 cells.

## Discussion

In this study, we characterized the status of PARylation and poly(ADP‐ribose) metabolism‐related enzymes in PARG inhibitor PDD00017273‐resistant HCT116R^PDD^ cells. (i) PARG protein levels were similar between parental HCT116 cells and resistant HCT116R^PDD^ cells. (ii) PARP protein levels were reduced in the resistant cells compared to the parental cells. (iii) Intracellular PAR levels were significantly elevated in the resistant cells relative to the parental cells. (iv) ARH3 protein levels were lower in the resistant cells than in the parental cells. (v) NAMPT protein levels were comparable in both cell lines. Based on these findings, we hypothesized that the resistant HCT116R^PDD^ cells, characterized by PAR accumulation and decreased PARP expression, might exhibit increased sensitivity to anticancer agents, irradiation, and PARP inhibitors compared to their parental counterparts. Therefore, the resistant HCT116R^PDD^ cells exhibited similar sensitivity to the anticancer drugs 5‐FU and CDDP as their parental HCT116 cells, despite elevated intracellular PAR levels, and were more sensitive to irradiation than the parental cells. This result may be attributed to the mechanism by which irradiation causes more direct DNA damage than 5‐FU or CDDP, thereby increasing intracellular PAR levels beyond tolerable thresholds in the resistant HCT116R^PDD^ cells. Excessive PARP activation and PAR accumulation are known to trigger cell death, including a specific form termed parthanatos [28, 29, 30]. Furthermore, despite the observed reduction in PARP1 expression, the resistant HCT116R^PDD^ cells did not display hypersensitivity to PARP inhibitors compared with the parental cells. These results suggest the presence of regulatory mechanisms in the resistant cells that prevent further increases in intracellular PAR levels.

It is well established that maintaining intracellular PAR levels is critical for cell survival and that excessive PAR accumulation can lead to NAD^+^ depletion and impaired ATP synthesis [25, 26, 27]. Functional inhibition of PARG has also been reported to reduce NAD^+^ levels when PAR accumulates [10, 26]. However, in our study, the resistant HCT116R^PDD^ cells exhibited intracellular NAD^+^, NADH, and ATP levels comparable to those of the parental HCT116 cells. Based on this finding, we hypothesized that NAD^+^ biosynthesis is upregulated in the resistant HCT116R^PDD^ cells. Therefore, we focused on the NAD^+^‐Nam *salvage* pathway, a key route for regulating intracellular PAR levels among NAD^+^ biosynthetic pathways. To explore this, we examined the sensitivity of both cell types to FK866, a specific inhibitor of NAMPT, the rate‐limiting enzyme in this pathway. Indeed, the resistant HCT116R^PDD^ cells showed increased sensitivity to the NAMPT inhibitor FK866 compared with their parental counterparts. These findings suggest that the resistant HCT116R^PDD^ cells may evade cell death and develop resistance to PDD00017273 by increasing reliance on the NAD^+^‐Nam salvage pathway and enhancing NAD^+^ biosynthesis, thereby preventing intracellular NAD^+^ and ATP depletion.

In conclusion, our study suggested that cancer cells possess mechanisms to evade cell death caused by PAR accumulation by sustaining intracellular NAD^+^ and ATP levels through PARP–PARG coregulation and modulating dependence on the NAM salvage pathway within the NAD^+^ biosynthesis process.

## Conflict of interest

The authors declare no conflict of interest.

## Author contributions

AS conceived and designed the study. KT, YO, and AS acquired the data. KT, YO, and AS analyzed and interpreted the data, and AS wrote the manuscript.

## Data Availability

The authors confirm that the data supporting the findings of this study is available within the article.
